# Comparative long-term outcomes of unicompartmental and total knee arthroplasty in knee osteoarthritis patients: a systematic review and meta-analysis

**DOI:** 10.3389/fsurg.2024.1405025

**Published:** 2024-08-21

**Authors:** Hai Hu, Pengfei Li, Zelin Liu, Hang Lv, Xiangjun Yang, Peiran Liu

**Affiliations:** ^1^ Department of Bone Injuries, Hanan Branch of the Second Affiliated Hospital of Heilongjiang University of Traditional Chinese Medicine, Harbin, China; ^2^ Department of Medical Services Division, The Second Affiliated Hospital of Heilongjiang University of Traditional Chinese Medicine, Harbin, China

**Keywords:** long-term outcomes, unicompartmental knee arthroplasty, total knee arthroplasty, meta-analysis, randomized controlled and cohort trials

## Abstract

**Background:**

Long-term outcomes for knee osteoarthritis patients undergoing unicompartmental knee arthroplasty (UKA) and total knee arthroplasty (TKA) remain inconclusive.

**Objectives:**

This study aims to evaluate the long-term outcomes over five years, including Knee Society Pain Scores (KSPS), Knee Society Scores (KSS), Knee Society Function Scores (KSFS), range of motion (ROM), and survival rates—of UKA vs. TKA in knee osteoarthritis patients.

**Design:**

Systematic review using data from randomized controlled and cohort trials, and world databases.

**Data sources:**

Researchers searched Medline, Embase, Cochrane Controlled Register of Trials, and ClinicalTrials.gov from January 1990 to March 2024.

**Eligibility criteria for selecting studies:**

The researchers selected studies based on adult participants with knee osteoarthritis. Eligible studies compare UKA and TKA reports on clinical or surgical outcomes, including KSPS, KSS, KSFS, ROM and survival rates, over 5 years. The researchers excluded the studies fewer than five years, or if English text was unavailable.

**Results:**

Researchers categorized twenty-nine eligible studies into three groups: five randomized controlled trials, 11 registries and database studies, and 13 cohort studies. The analysis revealed that neither TKA nor UKA definitively outperformed the other in terms of pain (SMD (95% CI): −0.06 [−0.41, 0.28], *I*^2 ^= 90%) and KSS scores (SMD (95% CI): −0.07 [−0.23, 0.008], *I*^2 ^= 81%) over a period of five years. However, KSFS (SMD (95% CI): −0.30 [−0.43, −0.17], *I*^2^ = 74%) and ROM (SMD (95% CI): −0.78 [−1.11, −0.46], *I*^2^ = 92%) tended to favor UKA, and survival rate favor TKA at 5 or over 5-year follow-up periods.

**Conclusions:**

UKA shows a trend towards better outcomes in KSFS and ROM, alongside a more favorable survival rate in TKA at the five-year and beyond follow-up periods.

**Systematic Review Registration:**

https://www.crd.york.ac.uk/prospero/display_record.php?RecordID=517835, PROSPERO (CRD42024517835).

## 1 Introduction

Knee osteoarthritis (OA) is a debilitating condition that significantly compromises the health-related quality of life (HRQoL) and functional status of afflicted individuals (1). Total knee arthroplasty (TKA) has emerged as the gold standard for treating severe OA, backed by substantial evidence supporting its efficacy in long-term outcomes and quality-of-life improvements, as measured through metrics like EQ-5D and WOMAC (2–4) Isolated medial OA is a form of knee arthritis that affects only the medial compartment of the knee joint, sparing the other compartments. In managing this condition, there is a debate between unicompartmental knee arthroplasty (UKA) and TKA, with both options providing successful outcomes but differing in functionality and patient expectations (5). UKA, on the other hand, offers a targeted surgical alternative for late-stage isolated compartment OA (6, 7). UKA should be considered the first choice for patients with late-stage isolated medial compartment OA due to its reliable outcomes (8). TKA often involves a more extensive surgical intervention that affects the whole knee, making it less suitable for isolated compartment OA. UKA holds several potential advantages over TKA, including less invasive surgical exposure, reduced morbidity and mortality, preservation of native bone stock, and the retention of cruciate ligaments. These features frequently translate into enhanced postoperative recovery and possibly a higher HRQoL (9).

Given the differential advantages and outcomes between UKA and TKA, the debate about the effects of both techniques are still continuing (10). UKA and TKA are critical surgical interventions for managing knee osteoarthritis, with TKA being the more prevalent procedure due to its applicability to extensive joint degeneration. However, UKA offers distinct advantages, such as preservation of healthy tissue, quicker recovery times, and improved functional outcomes for patients with localized knee arthritis (11, 12). Despite these benefits, the adoption of UKA remains limited, primarily due to concerns about long-term survivorship and a higher revision rate compared to TKA (13). Understanding these dynamics is essential for clinicians in making informed decisions and optimizing patient outcomes, necessitating a closer examination of current usage patterns and clinical evidence supporting each approach.

Researchers chose to investigate the long-term outcomes of UKA and TKA because existing literature predominantly focuses on short-term results, leaving a gap in understanding the enduring effects of these surgical procedures. Evaluating these interventions over a period extending beyond five years is crucial to fully capture their clinical and functional impacts, including long-term survivorship, complication rates, and sustained improvements in quality of life metrics such as pain scores, knee society scores (KSS), knee society function scores (KSFS), and range of motion (ROM). This long-term data is essential for optimizing patient outcomes, refining surgical practices, and providing patients with realistic expectations, ultimately leading to more personalized and effective treatment strategies for knee OA. The study aims to address discrepancies in the literature and evolving surgical techniques by conducting a comprehensive meta-analysis to determine which surgical approach, UKA or TKA, yields the most favorable long-term outcomes for various types of knee OA.

## 2 Methods

### 2.1 Data and literature search

The researchers searched for eligible English studies, including MEDLINE, EMBASE, Cochrane Library, and KoreaMed, dated from January 1990 to March 2024. The search was designed to capture all relevant studies comparing UKA and TKA, focusing on randomized controlled trials (RCTs), cohort studies, and registry data. The researchers conducted this study in accordance with the Cochrane Review Methods and the PRISMA (Preferred reporting items for systematic reviews and meta-analyses) (14).

The exclusion of non-English studies in this systematic review and meta-analysis is primarily due to practical considerations related to the language proficiency of the research team and the resources available for accurate translation. Including studies published in languages other than English would require extensive translation efforts, which could introduce errors and inconsistencies in data interpretation and synthesis. Additionally, the quality and availability of translations may vary, potentially leading to misinterpretation of the study findings and conclusions. While this exclusion criterion might limit the comprehensiveness of the analysis, it ensures that all included studies are thoroughly understood and accurately assessed, thereby maintaining the integrity and reliability of the review's conclusions. Furthermore, the predominance of high-impact medical research being published in English helps mitigate the impact of this exclusion, although it remains a limitation that should be acknowledged and addressed in future research with more robust multilingual resources.

### 2.2 Search terms

The researchers used a combination of keywords and MeSH terms tailored to maximize the retrieval of pertinent studies. The researchers employed the following search terms: “Total Knee Arthroplasty”, “Long-term outcomes”, “Unicompartmental Knee Arthroplasty”, “Scores of EU-5Q”, “pain scores”, “Knee Society Scores (KSS)”, “Knee Society Pain Scores (KSPS)”, “Knee Society Function Scores (KSFS)”, “Range of Motion (ROM)”, “Survival rate”, in English. To conduct a comprehensive electronic search in the MEDLINE database, Medline, Embase, Cochrane Controlled Register of Trials, and ClinicalTrials.gov, to identify relevant studies published in English between January 1990 and March 2024. The following search strategy could be used. This strategy incorporates the mentioned search terms and is designed to be replicable for consistency in the research process: (1) (“Total Knee Arthroplasty” [MeSH Terms] OR “Total Knee Arthroplasty” [All Fields] OR “TKA” [All Fields]); (2) (“Unicompartmental Knee Arthroplasty” [MeSH Terms] OR “Unicompartmental Knee Arthroplasty” [All Fields] OR “UKA” [All Fields]); (3) (“Long-term outcomes” [All Fields] OR “long-term effects” [All Fields] OR “long-term results” [All Fields]); (4) (“Survival rate” [MeSH Terms] OR “Survival rate” [All Fields] OR “Survival ship” [All Fields]); (5) (“Scores of EQ-5D” [All Fields] OR “EQ-5D” [All Fields]); (6) (“Pain scores” [All Fields] OR “pain assessment” [MeSH Terms] OR “pain measurement” [All Fields]); (7) (“Knee Society Scores” [All Fields] OR “KSS” [All Fields] OR “Knee Society Function Scores” [All Fields] OR “KSFS” [All Fields]); (8) (“Range of Motion” [MeSH Terms] OR “Range of Motion” [All Fields] OR “ROM” [All Fields]); (9) 1 OR 2; 10. 3 OR 4 OR 5 OR 6 OR 7 OR 8; 11. 9 AND 10.

### 2.3 Study selection

Two independent reviewers (HH and PL) performed the study selection based on title and abstract. In the case of any disagreement or uncertainty, full text was retrieved and reviewed, and discussed with a third reviewer (ZL). The randomized controlled and cohort trials, world databases or registries studies were included. TKA approaches were compared with UKA in these studies. In addition, they should report on at least one parameter related to clinical or surgical outcomes, such as perioperative pain, or ROM, or KSS, or KSPS, or KSFS, or Survival rate.

The decision to exclude studies with follow-up periods of less than 5 years is grounded in the need to comprehensively evaluate the long-term outcomes of knee OA interventions between UKA and TKA. A follow-up period of five years or more is considered effective for several reasons: Firstly, the five-year mark is commonly regarded as a benchmark for long-term clinical outcomes in medical research (15). Secondly, over a period of five years, it is possible to capture a comprehensive range of outcomes. This duration allows for the assessment of the durability of surgical benefits and the incidence of long-term complications or revision surgeries. Lastly, several studies report significant insights and trends in patient outcomes over a five-year period, making it a validated and reliable time frame for longitudinal studies. By adhering to these criteria, researchers can more accurately gauge the effectiveness of different surgical techniques in TKA. Eligible studies include participants with a confirmed diagnosis of knee OA graded as Kellgren–Lawrence Grade I or higher, or isolated compartment. The researchers will omit the studies with inadequate statistical methods. Secondly, studies main focus on the isolated patellofemoral joint or isolated lateral compartment osteoarthritis will also be excluded. The researchers excluded studies that lacked sufficient data for statistical pooling. Supplementary Table S1 provides a summary of all included studies and their key features.

### 2.4 Justification for exclusion of database studies

The exclusion of database studies from our meta-analysis was primarily driven by the substantial imbalance in sample sizes between studies, which poses a significant risk of bias and may skew the results. Here, we provide a more detailed justification. (1) Magnitude of Imbalance, the studies included in our meta-analysis vary significantly in terms of sample sizes (Supplementary Table S1). For instance, the study by Niinimaki et al. included 4,713 UKAs and 83,511 TKAs (16), whereas Gioe et al. had a sample size of only 738 TKAs and 127 UKAs (17). This substantial disparity in sample sizes can disproportionately influence the meta-analysis results, leading to overrepresentation of larger studies and underrepresentation of smaller ones. (2) Potential Bias, large registry-based studies often have different methodological approaches compared to smaller cohort studies. The differences in data collection, follow-up duration, and patient demographics can introduce heterogeneity that is not easily accounted for in a meta-analysis. This heterogeneity can affect the comparability of outcomes and lead to biased estimates. (3) Statistical Power and Precision, the large sample sizes in registry-based studies can lead to higher statistical power and more precise estimates of effect sizes. However, when combined with smaller studies, these large studies can dominate the overall meta-analysis, reducing the influence of smaller studies and potentially masking important findings.

### 2.5 Selection of domains of outcomes to be investigated

#### 2.5.1 Study characteristics

##### 2.5.1.1 Study design

Identifying whether the study is randomized, observational, or uses another design.

##### 2.5.1.2 Follow-up

The duration of postoperative follow-up, essential for assessing long-term outcomes.

##### 2.5.1.3 Sample size

Total number of participants, which impacts the study’s statistical power.

#### 2.5.2 Participant demographics and baseline characteristics

##### 2.5.2.1 Propensity matched

Indicates if the study used propensity score matching to balance baseline characteristics.

##### 2.5.2.2 Inclusion criteria

Specific criteria used to enroll participants, defining the study population.

##### 2.5.2.3 Mean age (SD or range)

Average age of participants, with standard deviation (SD) or range, to understand the age distribution.

##### 2.5.2.4 Sex (M:F)

Gender distribution, presented as a ratio of males to females.

##### 2.5.2.5 Mean BMI (SD)

Average Body Mass Index (BMI) of participants, with SD, to assess weight-related factors.

##### 2.5.2.6 Kellgren–Lawrence scale

Radiographic grading of knee osteoarthritis severity.

##### 2.5.2.7 OA category (isolated compartment, yes or not)

Whether osteoarthritis is confined to a single compartment of the knee.

#### 2.5.3 Clinical outcomes

##### 2.5.3.1 Bristol knee score

Patient-reported measure of knee function and pain.

##### 2.5.3.2 Knee society pain score

Evaluation of knee pain severity.

##### 2.5.3.3 Knee society function score

Assessment of functional activities.

##### 2.5.3.4 Knee society score

Combined measure of knee function and pain.

##### 2.5.3.5 ROM (range of motion)

Degree of knee flexion and extension post-surgery.

##### 2.5.3.6 Surgical complications

Incidence and type of postoperative complications.

##### 2.5.3.7 Hospital stay

Length of hospitalization post-surgery.

##### 2.5.3.8 Operation time (min)

Duration of the surgical procedure.

##### 2.5.3.9 Survival rate

Proportion of implants surviving without revision at follow-up.

### 2.6 Data extraction

The researchers used a predefined data extraction form for data extraction. Two reviewers (HL and XY) independently extracted data related to Study Design, Follow up, Sample Size, Propensity Matched, Inclusion Criteria, Mean Age (SD or range), Sex (M:F), Mean BMI (SD), Kellgren–Lawrence Scale, OA Category (Isolated compartment, yes or not), KSPS, KSFS, KSS, ROM, Surgical Complications, Hospital Stay, Operation Time (min), Survival Rate, and Other Outcomes over a period of five years or longer. The researchers removed duplicated literatures. The reviewers resolved disagreements by reaching a consensus or consulting a third investigator (PL). Detailed information about the surgical techniques, prosthetic models used, and specifics of physiotherapy treatments might not be explicitly stated. It's assumed that the interventions were standardized across the studies to some extent, or that variability in these factors was considered in the analysis. The document does not detail the specifics of postoperative care, including physiotherapy regimens and follow-up protocols. It's assumed that there was a general standard of care followed across the studies, which could impact recovery and long-term outcomes. The researchers categorized the results into three groups: randomized controlled and cohort trials, and world database or registry studies (Supplementary Table S1).

### 2.7 Patient and public involvement

Incorporating patient and public perspectives was a pivotal aspect of our research process. Patient discussion groups played a key role in shaping the research question and determining relevant outcome measures, reflecting a commitment to patient-centered research. However, it's important to note that patients did not participate in interpreting the study results or in the manuscript preparation. Furthermore, plans for disseminating the research findings do not currently include direct patient involvement. This approach underscores our belief in the importance of patient input in the early stages of research while maintaining a traditional framework for analysis, interpretation, and dissemination of results.

### 2.8 Assessment of methodological quality

The researchers assessed the methodological quality through a risk of bias table and the modified Jadad scale. Randomization procedure, allocation concealment, blinding, selective outcome reporting, and incomplete outcome data were among the assessed criteria. Two reviewers participated in the assessment process. This dual-reviewer approach is standard in systematic reviews and meta-analyses to minimize subjective bias and enhance the reliability of the evaluation process. The reviewers worked independently to assess each study's methodological quality. This independent assessment ensures that each reviewer's conclusions are reached without influence from the other, thereby reducing the risk of bias in the evaluation process itself. While it was not explicitly mentioned, the standard procedure in such reviews involves resolving disagreements between reviewers through discussion or consultation with a third investigator. This step is crucial to reach a consensus on the methodological quality of each study and to ensure that the risk of bias assessment is as accurate and unbiased as possible.

### 2.9 Data synthesis and analysis

The researchers focused on clinical outcomes and clinical metrics such as KSPS, KSS, KSFS, and ROM as the primary interests, and evaluated statistical heterogeneity among the included studies using Q statistics and *I*^2^ test. Depending on the *I*^2^ value, the researchers applied a fixed or random effect model. The researchers conducted analysis using RevMan version 5.2 software.

The researchers addressed data inconsistencies by contacting original authors for missing summary statistics and, if unsuccessful, using imputation techniques or sensitivity analyses. They standardized outcomes into a common metric for direct comparisons, calculating standardized mean differences for continuous outcomes or converting odds ratios to risk ratios for dichotomous outcomes. Heterogeneity was assessed to select the appropriate meta-analysis model, and subgroup analyses were planned to explore variations. To analyze KSFS and ROM trends between preoperative and postoperative periods for UKA and TKA, improvements were calculated by subtracting preoperative from postoperative values. Descriptive statistics and paired *t*-tests compared values within each group, while two-sample *t*-tests compared improvements between TKA and UKA. *P*-values and confidence intervals were reported to determine statistical significance. For studies without exact preoperative data but showing non-significant baseline differences, recent postoperative data were used to ensure robust analysis of UKA and TKA effectiveness in KSFS and ROM improvements.

### 2.10 Risk of bias assessment

Researchers used the Cochrane Collaboration's tool to evaluate the risk of bias in randomized controlled trials, categorizing each study as high, unclear, or low risk across several dimensions such as random sequence generation, allocation concealment, blinding, and attrition bias (Supplementary Table S2). For cohort, database, and registry studies, the Newcastle-Ottawa Scale assessed selection, comparability, outcome assessment, and follow-up, with risks similarly classified and summarized in Supplementary Tables S3, S4. Two additional reviewers (HL and PL) participated in the assessment process to ensure methodological quality.

### 2.11 Statistical analysis

The main authors (HH, PL, ZL) performed the statistical analysis using an inverse variance weighted random effects model to calculate overall summary estimates for each outcome. This method accommodates anticipated heterogeneity, quantified using the *I*^2^ statistic and Q test, and results were visually presented in forest plots showing individual and summary relative risk estimates. To address significant methodological differences, the data were stratified by study type (randomized controlled trials, registry studies, and large cohort studies) for clarity. When specific outcome data were missing, researchers provided comments to address these gaps, ensuring a comprehensive analysis.

## 3 Results

### 3.1 Clinical baseline characters

**Figure 1:**
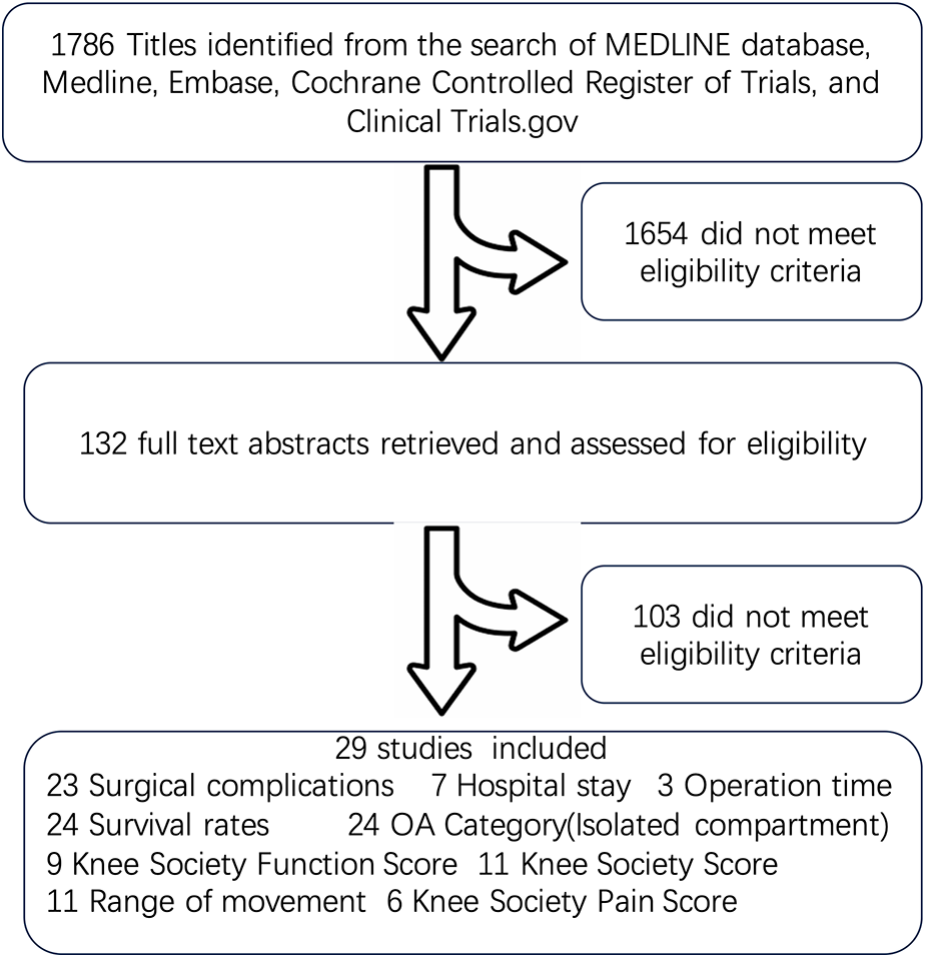
Flow diagram of literature search and study selection for meta-analysis comparing long-term outcomes (≥5 years) of unicompartmental and total knee arthroplasty. This flow diagram outlines the search and selection process for the meta-analysis focused on comparing the long-term outcomes (more than or equal to five years) of Unicompartmental Knee Arthroplasty and Total Knee Arthroplasty. The diagram depicts the initial database searches, screening of abstracts and titles, full-text assessments, and the reasons for exclusions.

The initial literature search resulted in 1,786 articles, of which 1,654 did not match the eligibility criteria and a further 103 did not match the eligibility after full text reviewing. In the end, this left 5 RCT (444 TKA and 448 UKA) (8, 18–21), 13 cohort trial (13,592 TKA and 1,915 UKA because Craik et al. reported unmatched cases: 546 UKA and 6,753 TKA, and Lyons et al. reported unmatched cases: 5,606 TKA and 279 UKA) (22–33) and 11 database and registry studies (268,376 TKA and 26,579 UKA) (16, 17, 34–42) for inclusion (Figure 1; Supplementary Table S1). The age of participants in both TKA and UKA groups is comparable across studies, with minor differences in average ages that are unlikely to be clinically significant. This suggests that the outcomes are not biased by age differences between groups (Supplementary Table S1). The Kellgren–Lawrence grading scale shows that both TKA and UKA groups were dealing with similar severities of osteoarthritis in five studies (18–20, 30, 32). The data indicate that the TKA and UKA groups are broadly comparable in terms of gender distribution, age, OA severity, and categories. However, there are differences in surgical complications and survivorship rates, which might suggest that while the groups are comparable, the outcomes can vary depending on the type of surgery.

### 3.2 Pain management of UKA vs. TKA

**Figure 2:**
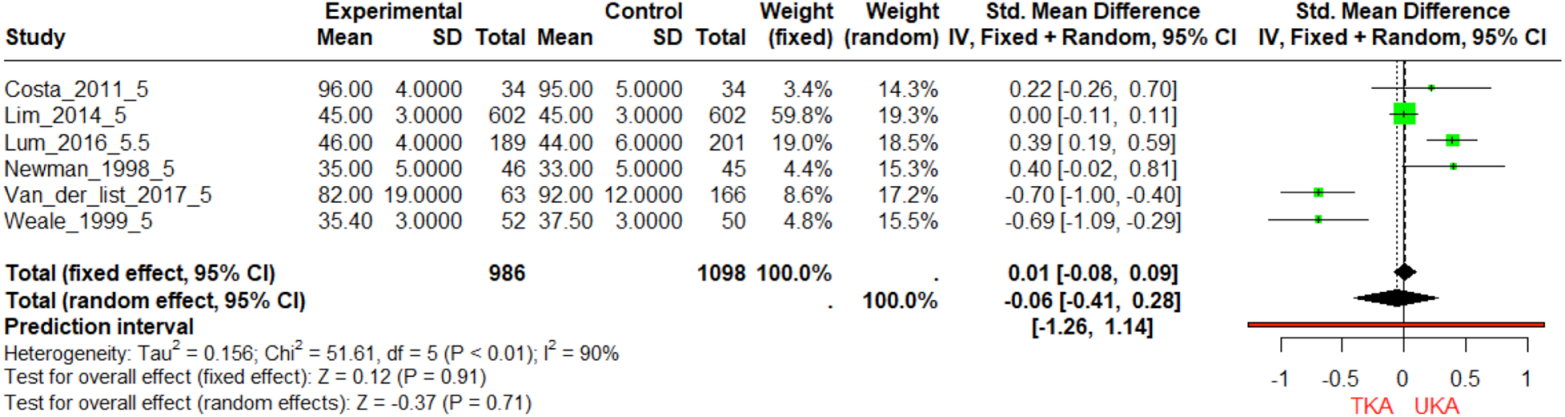
Forest plot of standardized mean differences (SMD) in 5-year pain scores between TKA and UKA groups. The forest plot depicts the SMDs in 5-year follow-up pain scores between TKA and UKA groups across three studies. Each horizontal line represents a study with its corresponding 95% confidence interval. The square marker indicates the SMD, and its size is proportional to the weight of the study in the meta-analysis. The diamond at the bottom represents the pooled SMD, calculated using both fixed and random-effects models.

A 5-Year Follow-Up Pain Score for TKA and UKA Groups: In the investigation conducted by Newman et al., the TKA group had a mean 5-year pain score of 35, whereas the UKA group recorded a mean pain score of 33 (20). The calculated standardized mean difference (SMD) was 0.40, which was not statistically significant with a *p*-value of 0.061 (Figure 2). In contrast, Weale's research in 1999 reported that the TKA group had a mean 5-year pain score of 35.4, and the UKA group had a mean score of 37.5 (21). The SMD was −0.69, which was statistically significant with a *p*-value less than 0.001 (Figure 2). The study by Lim et al. (2014) is particularly intriguing as it found absolutely no difference in 5-year pain scores between the TKA and UKA groups, both having a mean score of 45 (33), resulting in an SMD of 0 with a *p*-value of 1. Considering the three studies together, there is a high level of heterogeneity as indicated by an *I*^2^ value of 90%. These studies do not present a clear advantage of one procedure over the other.

### 3.3 Knee scores of UKA vs. TKA

**Figure 3:**
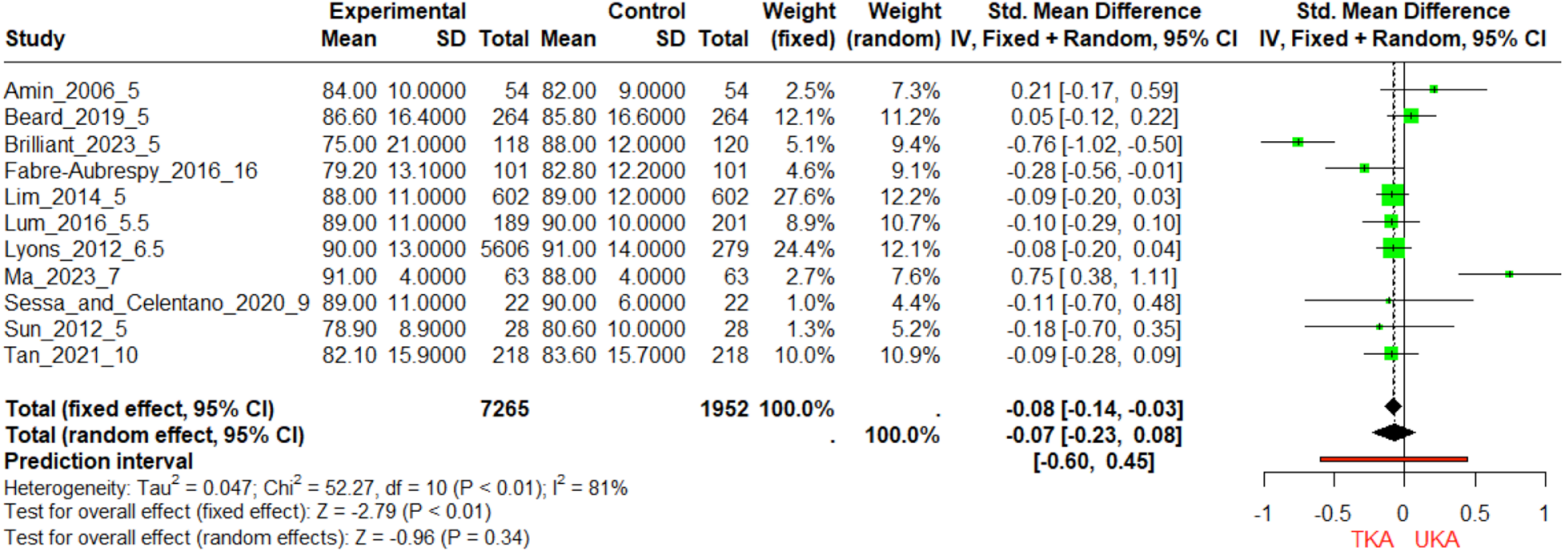
Forest plot of standardized mean differences (SMD) in KSS across multiple follow-up periods for TKA and UKA groups. The forest plot visually synthesizes the SMDs in KSS scores from studies conducted at 5-year, 10-year, and 16-year follow-up periods. The bottom diamond marker provides the pooled SMD, based on both fixed and random-effects models.

At the 5-year follow-up, UKA shows a slightly higher or comparable mean KSS to TKA, with Brilliant et al. reporting a notably higher mean for UKA (88 vs. 75) (Figure 3). The SMDs are small and close to zero, indicating minimal differences, and Beard et al. shows only a minor KSS difference despite a large sample size. The overall pooled effect size slightly favors UKA (−0.08 for fixed effects). Between 5.5 and 10 years, KSS differences remain minimal, with Lyons et al., Sessa and Celentano, and Lum et al. reporting similar means for both procedures, though Tan et al. shows a slightly higher mean for UKA. The trend suggests UKA maintains a slight medium-term advantage. At 16 years, Fabre-Aubrespy et al. shows a higher mean KSS for UKA (82.8) vs. TKA (79.2), indicating a small but persistent advantage, though the overall effect is not significant (−0.07 for random effects).

**Figure 4:**
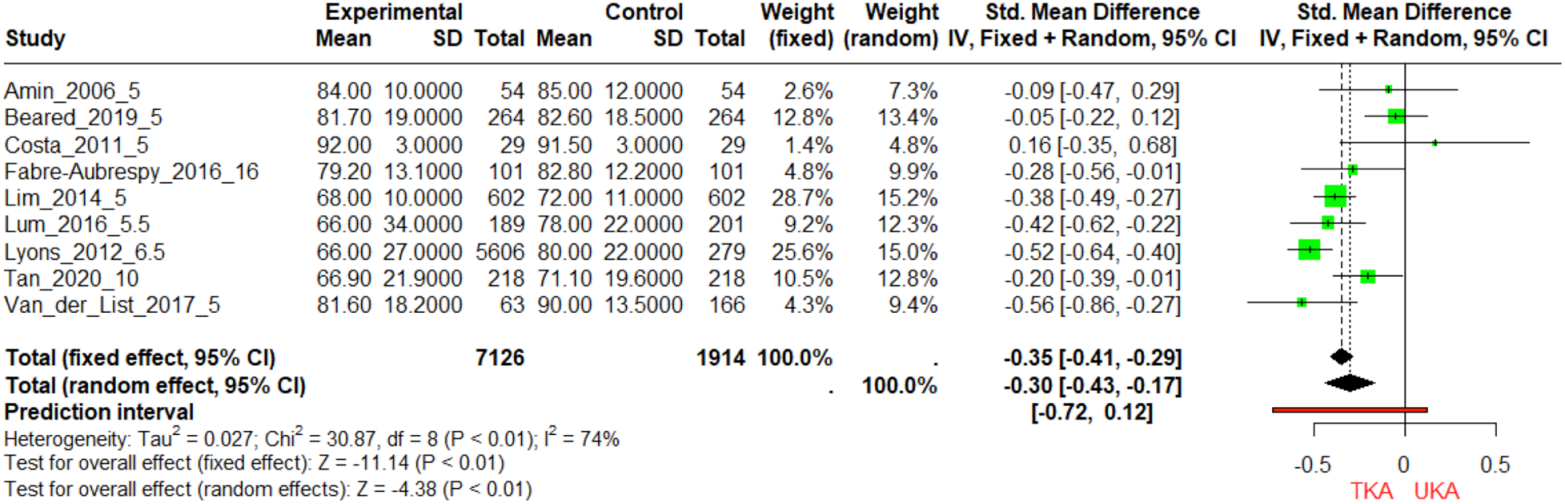
Comparative analysis of knee society function scores (KSFS) in TKA and UKA groups over different follow-Up periods. The figure portrays the Standardized Mean Differences (SMDs) in KSFS for TKA and UKA groups across three-time intervals: 5, 10, and 16 years. Each horizontal line symbolizes the confidence interval of each study, and the diamond marker specifies the SMD.

At the 5-year mark, studies by Amin (18) and Costa (43) show equivalent sample sizes for TKA and UKA, with UKA having a slightly higher mean KSFS (Figure 4). Lum and Lyons also report higher mean KSFS for UKA but with more variability. Van der List et al. show UKA with a significantly higher mean KSFS of 90 compared to 81.6 for TKA and a smaller standard deviation, indicating consistent outcomes. Between 5.5 and 10 years, all three studies report higher mean KSFS for UKA, with Tan et al. showing a substantial 4.2-point difference. Lyons et al., with the largest sample size (5,606 for TKA and 279 for UKA), reports a 14-point difference in favor of UKA, significantly impacting the meta-analysis. Lum et al. show a 12-point difference with high variability for TKA. At the 16-year follow-up, Fabre-Aubrespy et al. report a higher mean KSFS for UKA by 3.6 points, with similar standard deviations for both groups, suggesting that UKA may yield slightly better long-term functional outcomes, although this conclusion is based on a single study.

The comparison of KSFS improvements between TKA and UKA shows mixed results (Supplementary Table S5). Studies by Fabre and Beard indicate no significant difference between the two groups (*p* > 0.05), with UKA showing slightly better improvements but not to a statistically significant extent. The Amin study also shows no significant difference (*p* > 0.05), with TKA having a marginally higher improvement (18). However, studies by Tan, Van, and Lyon show statistically significant improvements favoring UKA (*p* < 0.05), with UKA demonstrating larger improvements in KSFS compared to TKA. Specifically, Tan's study shows an improvement of 37.5 ± 16.6 for UKA compared to 35.5 ± 16.8 for TKA (29), and Van's study reports an improvement of 34.8 ± 4.5 for UKA vs. 27.9 ± 5.8 for TKA (28). Lyon's study also supports UKA with a significant improvement difference (27).

### 3.4 Better ROM following UKA

**Figure 5:**
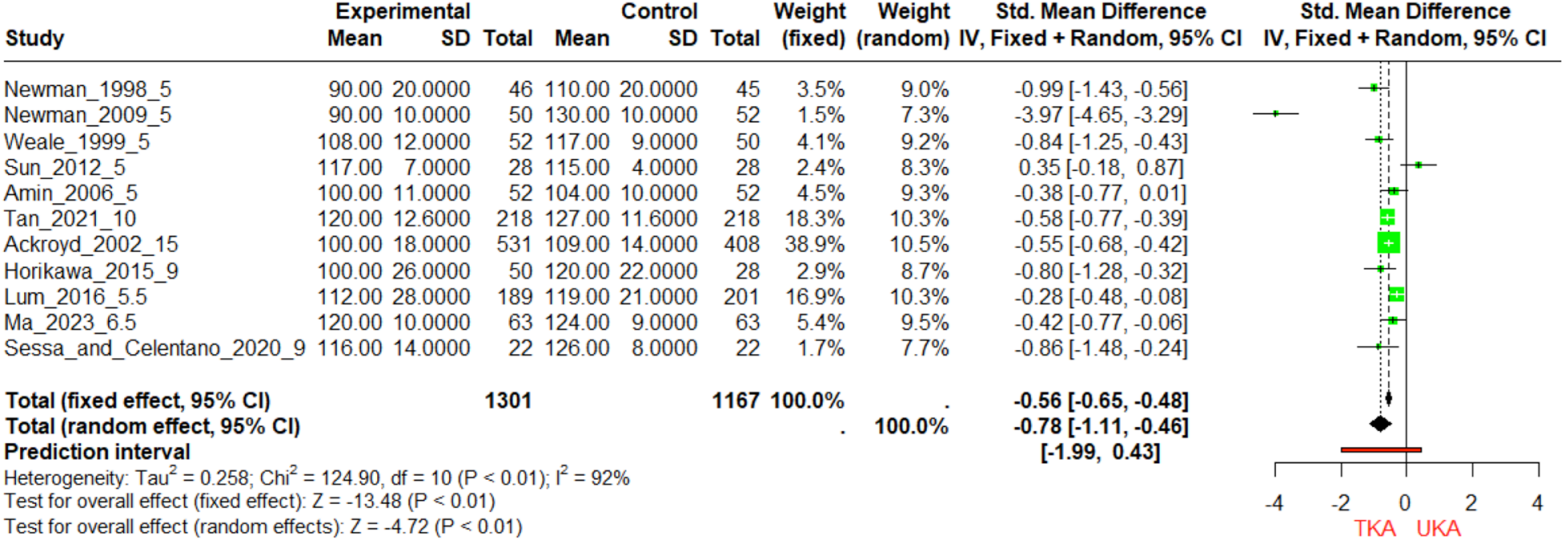
Forest plot of standardized mean differences (SMD) in 5-year and 10-year follow-up ROM scores between TKA and UKA groups this forest plot illustrates the SMDs in range of motion (ROM) scores for TKA and UKA groups at 5-year and 10-year follow-up periods. The size of each square marker is proportional to the weight (“w.fixed” or “w.random”) of the study in the meta-analysis.

During the 5-year follow-up, UKA is designed to preserve more of the natural knee mechanics. Newman et al. (19) observe the largest mean difference, with the UKA group scoring a mean of 130 compared to the TKA group's 90, which indicates a significantly better ROM for UKA. Similarly, in Newman et al. (20), the UKA group has a mean score of 110 compared to the TKA's 90. Across these 5-year studies, UKA demonstrates superior ROM outcomes (Figure 5).

For the 5.5–10-year follow-up, UKA generally provides better ROM than TKA. Lum et al. reports a UKA mean of 119 vs. TKA's 112. Horikawa_2015 shows a more pronounced difference with UKA's mean at 120 against TKA's 100. In Ma_2023, the means are closer, with UKA at 124 and TKA at 120. These results are indicative of the preservation of mobility with UKA, with the forest plot again showing negative SMDs, suggesting that UKA is favorable for maintaining a greater ROM. At the 15-year mark, the data from Ackroyd et al. shows that UKA has a mean ROM score of 109 compared to TKA's 100. While the advantage for UKA seems to have narrowed over time, it still remains the favorable procedure for ROM according to this data set. In summary, the forest plot and the accompanying data table suggest that patients undergoing UKA tend to have better ROM outcomes compared to those undergoing TKA. The difference is most notable in the short-term (5 years) and seems to persist, although possibly to a lesser extent, into the long-term (up to 15 years). The negative values in the SMD column in the forest plot support the conclusion that UKA is superior to TKA in terms of ROM, as higher ROM scores indicate better knee flexibility and function.

UKA generally demonstrates better improvements in ROM compared to TKA (Supplementary Table S5). The Weale study shows a significant improvement for UKA (117 ± 9.4) compared to TKA (*p* < 0.05) (21). In the Tan study, UKA shows a significant improvement (2.0 ± 5.5) compared to TKA (−4.3 ± 2.6) (*p* < 0.05) (29). The Admin study also favors UKA with an improvement of 2 (0.3) over TKA's −5 (0.7) (*p* < 0.05) (18). Newman 1998 indicates a substantial advantage for UKA with an improvement of 54.8% > 120 over TKA's reduction −1.9% > 120 (*p* < 0.05) (20). Additionally, Newman 2009's referred conclusion in the paper indicates significant improvement for UKA (*p* < 0.05) (19). Non-significant improvements are noted in the Ackroyd study with TKA improving by 9.0 (1.8) and UKA by 5.5 (0.9) (*p* > 0.05) (22), and the Horikawa study shows TKA at −9—(−0.7) and UKA at −4—(−0.5) (*p* > 0.05) (25). The Sun, Lum, Sessa, and Ma studies also report non-significant differences (*p* > 0.05) (26, 32, 44). Overall, the trend suggests that UKA tends to have a more favorable outcome in terms of ROM improvements where significant differences are observed.

### 3.5 Higher level of survival rates following TKA

**Figure 6:**
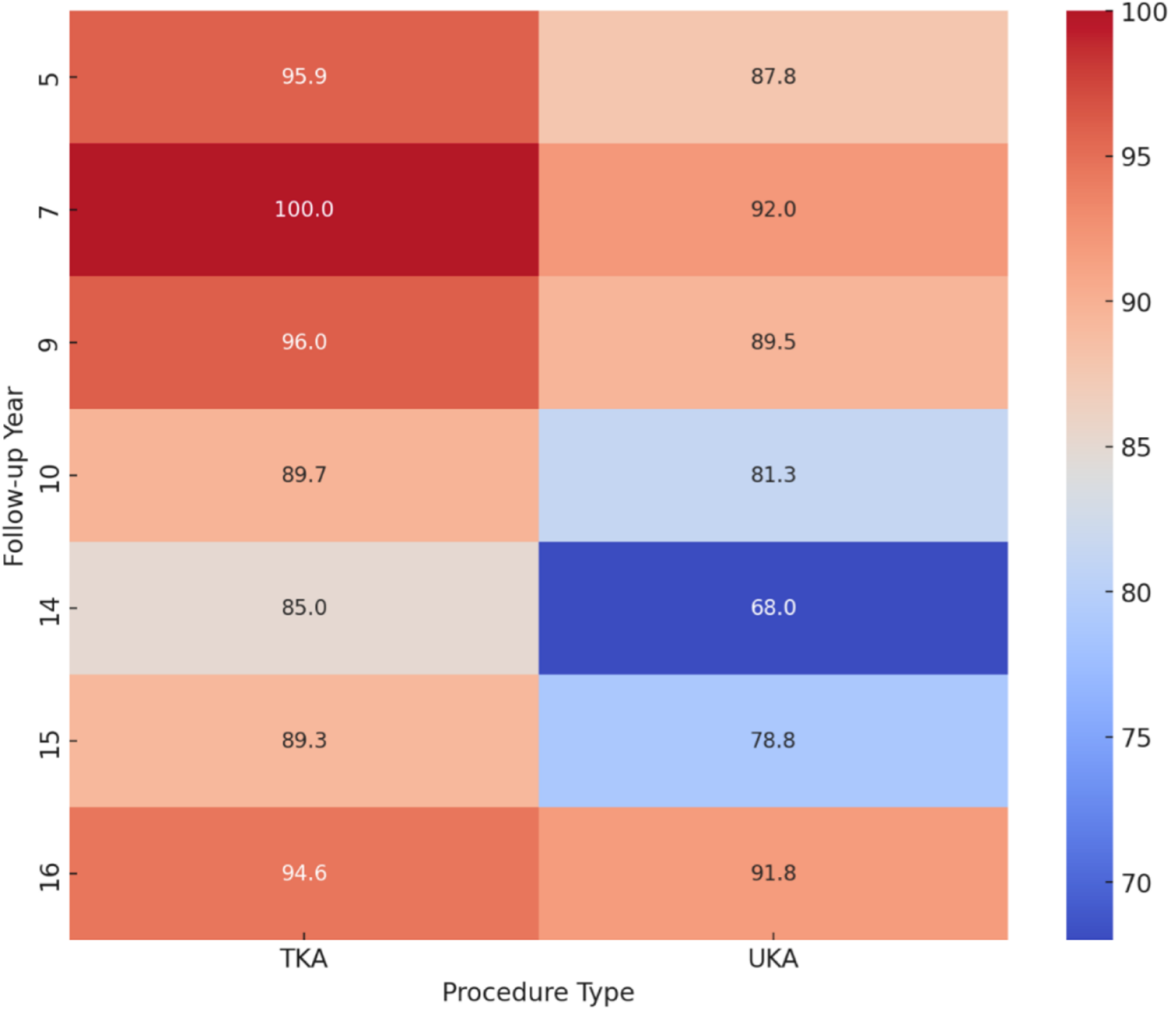
Average survival rate heatmap of total knee arthroplasty (TKA) and unicompartmental knee arthroplasty (UKA) across varying follow-up durations. The horizontal axis represents the type of knee arthroplasty procedure (TKA or UKA), while the vertical axis delineates the follow-up years post-surgery. The color gradient reflects the survival rate percentage, with warmer colors indicating higher survival rates and cooler colors denoting lower rates.

The comparative heatmap elucidates the divergence in survival rates between TKA and UKA across various follow-up years, highlighting the longitudinal efficacy and durability of these surgical interventions. Notably, TKA consistently exhibits superior survival rates over UKA, emphasizing TKA's robustness as a knee arthroplasty treatment (Figure 6). At the 5-year follow-up, TKA shows a high survival rate averaging 97.5%, compared to UKA's 90%. This trend continues into extended periods, with TKA maintaining an 89% survival rate at 15 years, vs. 70% for UKA, underscoring TKA's long-term reliability. The heatmap also indicates a gradual decline in survival rates for both procedures over time, reflecting natural wear and potential complications. However, UKA's decline is more pronounced, suggesting a faster deterioration rate or higher complication incidence requiring revision surgery. The differential in survival rates is stark in longer follow-ups, such as the 14–15-year range, where TKA's survival rate remains significantly higher than UKA's. This analysis underscores the importance of considering long-term outcomes in knee arthroplasty decisions, favoring TKA for its durability and sustained performance, especially for patients likely to challenge their knee replacement's longevity.

### 3.6 Risk of bias for each included study

**Figure 7:**
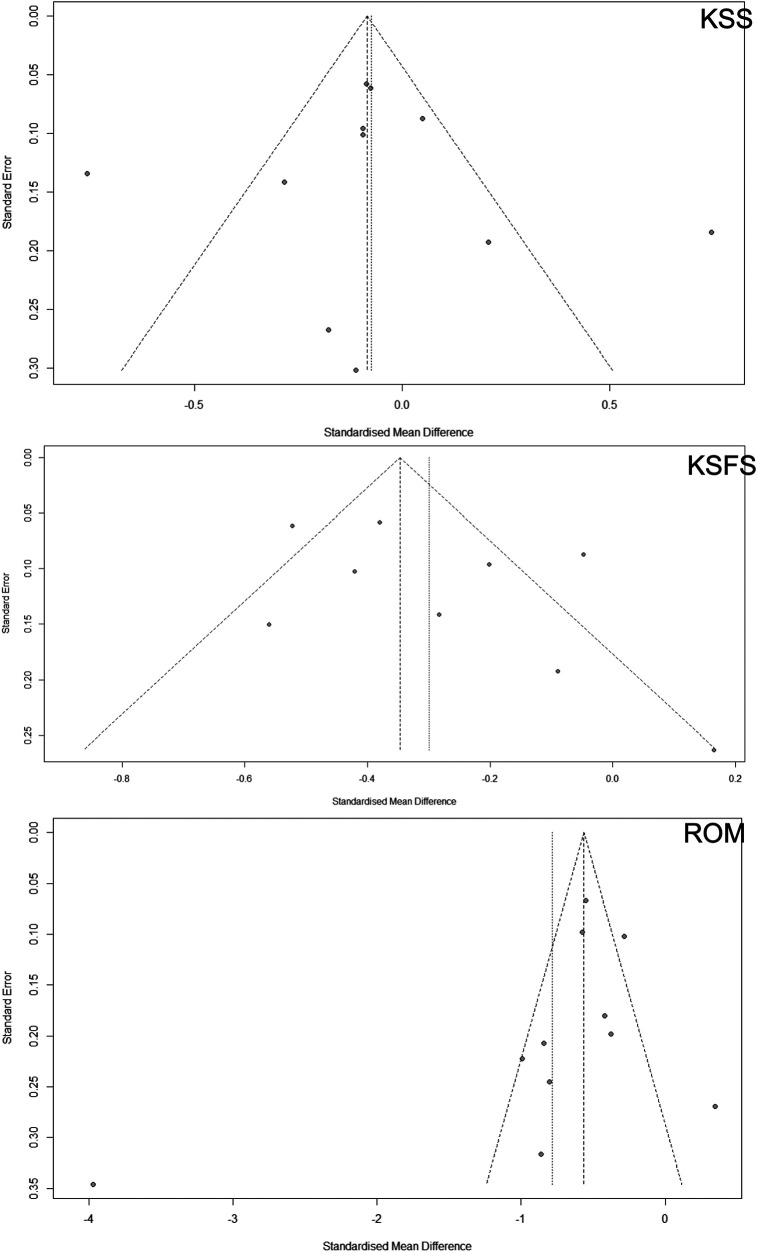
The potential publication bias in meta-analyses comparing total knee arthroplasty (TKA) and unicompartmental knee arthroplasty (UKA). Three clinical outcome parameters include Range of Motion (ROM), Knee Society Score (KSS), and Knee Society Functional Score (KSFS). Ideally, a symmetric distribution around the mean effect size line would suggest minimal bias, but asymmetry could indicate a tendency toward publishing studies with more significant or positive results.

For the KSS funnel plot, the distribution of studies appears relatively symmetrical around the vertical line that represents the pooled effect size, suggesting minimal publication bias for this particular outcome (Figure 7). Both Brilliant (30) and Ma (32) are considered underrepresented studies in the funnel plot due to their relatively smaller sample sizes and larger standard errors, positioning them outside the main triangular funnel area (Figure 7). For Brilliant, the confidence interval is entirely below zero, indicating a strong negative effect size (*p* < 0.05) (30). For Ma, the confidence interval is entirely above zero, indicating a strong positive effect size (*p* < 0.05) (32). The KSFS funnel plot shows a noticeable asymmetry, with fewer studies reporting negative standardized mean differences than expected. This asymmetry implies potential publication bias, where studies with smaller sample sizes and negative effect sizes are possibly unpublished or harder to locate (18). Van der List is likely underrepresented in the funnel plot due to its positioning outside the main triangular funnel region and higher standard error (28). For Van der List, the confidence interval is entirely below zero, indicating a strong negative effect size (*p* < 0.05) (28). Lastly, the ROM funnel plot displays pronounced asymmetry similar to that of the KSFS plot, with a dearth of studies reporting negative effect sizes. This is a clear indication of publication bias, particularly concerning smaller studies that are expected to be scattered at the bottom of the funnel. The absence of such studies suggests that negative or non-significant findings are also less likely to be published or included in the meta-analysis. The study by Newman has an effect size of −3.97, which is far from the pooled effect size and towards the extreme left of the plot (Figure 7) (19). For Newman, the confidence interval is [−4.65, −3.29] and entirely below zero, also indicating a strong negative effect size (*p* < 0.05) (19).

Analyzing the risk of bias in RCTs between TKA and UKA using the Cochrane collaboration's tool revealed varied rigor across studies (Supplementary Table S2). Beard et al. showed the lowest risk of bias (8), while Newman J et al. (19, 20) and Costa et al. (43) had concerns with sequence generation and selective outcome reporting. Weal et al. displayed unspecified biases (21). Cohort studies assessed with the Newcastle-Ottawa Scale showed consistent quality in selection and outcome measures (“a” rating) but concerns with comparability (“b” rating) (Supplementary Table S3). Ackroyd et al. (22), Amin et al. (18), and Lyons et al. (27) had “c” ratings in some areas, while Sessa and Celentano (31) received “a” ratings across all categories. Registry studies also showed robust quality in selection and outcomes (“a” rating) but had comparability concerns (“b” rating) and occasional follow-up issues (“d” rating) (Supplementary Table S4). Overall, while data quality is high, variability in comparability and follow-up may introduce bias and affect the validity of conclusions about TKA and UKA effectiveness.

## 4 Discussion

The 5-year follow-up studies on pain scores for TKA and UKA groups yield inconclusive results. While Weale's 1999 study suggests a statistically significant advantage for the TKA group (21), with lower pain scores, Newman's 1998 study shows no such advantage (20), reflected in a non-significant *p*-value of 0.061. Adding complexity to the narrative, Lim et al.'s 2014 study finds identical mean pain scores for both groups (33), resulting in a non-significant SMD with a *p*-value of 1.

The analyses of KSS scores at different time frames present an intricate landscape. In the short term, specifically at the 5-year follow-up, Newman et al.'s study found the TKA group to have lower KSS scores, substantiated by a highly significant *p*-value (20). Yet, other studies at the same follow-up period, such as those by Costa et al. and Sun et al., found no significant difference (43). The long-term view also varies: at the 10-year mark, Tan et al.'s data showed no statistically significant difference between TKA and UKA groups (29). Interestingly, at the 16-year follow-up, Fabre-Aubrespy et al. observed a significant advantage for the UKA group (24). The meta-analysis data reveals nuanced results based on the duration of follow-up. At the 5-year mark, Lim et al. find a significant advantage for UKA over TKA in KSFS, supporting its utility for better functional outcomes in the short-term (33). However, the benefit seems to taper off in the longer term, with the 16-year follow-up study by Fabre-Aubrespy et al. showing a non-significant trend favoring UKA (24). The SMDs are statistically significant in most cases, reinforcing the efficacy of UKA in providing better ROM outcomes (Figure 5). Postoperative assessments revealed that UKA patients experienced significantly improved ROM compared to TKA patients (9).

In terms of functionality, the values for minute walk test (2MWT) and Timed Up-and-Go test (TUG) at 1 and 2 years were similar after UKA and TKA (45). The researchers assessed postoperative pain with a 10 cm VAS. The difference with regard to VAS between the two groups could not show significance (44). Brown, Nicholas M., et al.'s seminal research over a 5-year follow-up period analyzed 2,235 TKA and 605 UKA patients, finding significantly higher postoperative complications for TKA at 11.0% compared to 4.3% for UKA. The study emphasizes TKA's elevated risks, including manipulation, transfusion, ICU admission, and longer hospital stays, suggesting the need for careful patient counseling and surgical decision-making (46). Hansen et al. conducted a comprehensive long-term comparative analysis of UKA and TKA using Medicare and MarketScan databases, covering up to 10 years post-surgery. The study found that while UKA patients had fewer postoperative complications and hospital re-admissions, they faced a significantly higher rate of re-operation and revision surgeries in the long term, with age being a critical risk factor for complications and implant failure (47).

The comparative heatmap analysis between TKA and UKA shows TKA's superior survival rates, with 97.5% at five years and 89% at 15 years, compared to UKA's 90% at five years and 70% at 15 years. This trend underscores TKA's robustness and long-term efficacy, making it the more durable option, particularly for patients with higher longevity demands on their knee replacements. Niinimäki et al. provided a long-term analysis from a 27-year dataset in the Finnish Arthroplasty Register, revealing that UKA has significantly lower long-term survivorship rates compared to TKA, with 15-year survivorship rates of 69.6% for UKA and 88.7% for TKA, based on samples of 4,713 and 83,511 patients, respectively. This comprehensive study underscores the higher risk of revision for UKA, despite its advantages, offering valuable insights for clinicians and patients in knee arthroplasty decisions (16).

The funnel plot assessment reveals that while the KSS data show less publication bias, the KSFS and ROM outcomes indicate potential bias, potentially distorting meta-analysis effect estimates and overestimating TKA and UKA efficacy. Addressing this bias is essential for ensuring the meta-analysis's integrity and providing an accurate evidence base for clinical decisions. Methods such as Egger's test are recommended for further validation. Enhanced KSFS and ROM in UKA patients lead to significant improvements in functional activities and knee flexibility, crucial for daily living and mobility. These improvements suggest that UKA may offer more natural knee movement, less postoperative pain, and faster recovery, benefiting younger, more active patients needing a quicker return to normal activities. Therefore, clinical decisions between UKA and TKA should consider these factors, tailoring the surgical approach to the patient's specific needs and lifestyle demands to enhance satisfaction and long-term outcomes.

With relatively balanced numbers (444 TKA vs. 448 UKA), the RCTs provide strong, controlled evidence, reducing biases and allowing for reliable comparisons between TKA and UKA. This balanced approach enhances the power to detect differences in outcomes, such as complications or recovery times, and is suitable for establishing causality. This vast difference allows for more robust statistical analyses and more reliable extrapolations of the data to real-world settings. Cohort studies, such as those by Craik et al. (23) and Lyons et al. (27), highlight the potential for selection bias in non-randomized settings but still provide valuable insights into real-world outcomes.

Larger samples in registry studies are crucial for detecting rare adverse events and understanding procedure-specific risks, which are vital for patient selection and surgical decision-making. Large-scale data, especially from registries, shape public health policy and clinical standards due to their broader applicability. To address imbalanced sample sizes without excluding valuable large database studies, we considered several methods: Weighted Analysis (studies weighted by inverse variance), Meta-regression (exploring relationships between study characteristics and outcomes), Subgroup Analysis (separating studies by sample size or design), and Sensitivity Analysis (including/excluding large studies to assess impact). Despite these methods, we excluded database studies from our meta-analysis to ensure robustness and reliability. The significant disparities in sample sizes and methodological differences between registry-based and smaller cohort studies could introduce biases that are not easily accounted for. By focusing on studies with comparable sample sizes, we aim to provide more accurate and meaningful insights into knee arthroplasty outcomes.

UKA is particularly suitable for older, less active patients with isolated compartmental OA due to its less invasive nature, reduced morbidity, and quicker recovery, preserving natural knee function (Figure 5). Conversely, TKA is more appropriate for younger, more active individuals or those with diffuse OA, as it provides a comprehensive solution for extensive joint damage and is robust and durable for substantial joint demands (Figure 6). The impact of study design on the outcomes of knee arthroplasty procedures, such as UKA and TKA, is significant and multifaceted. RCTs provide high-quality evidence by minimizing biases and allowing for reliable comparisons due to their controlled environments and balanced participant numbers (e.g., 444 TKA vs. 448 UKA) (48). This design enhances the ability to detect differences in outcomes like complications and recovery times, thereby establishing causality effectively. However, the smaller sample sizes in RCTs limit the generalizability of findings (45). The multifaceted approach is essential for developing evidence-based recommendations and personalized treatment strategies in knee arthroplasty (49). The variability in outcomes is influenced by surgical expertise, implant design, and patient selection, with surgeon proficiency significantly impacting success and complication rates. Advances in implant design also contribute to improved biomechanics and longevity, affecting survival rates and functional scores.

The systematic review and meta-analysis compared clinical outcomes of UKA vs. TKA. It included various outcomes such as pain VAS, KSF, ROM, complications, and revision surgery rates. The study found that UKA had fewer postoperative complications but higher revision rates compared to TKA over short-term follow-up periods (50). Another meta-analysis reviewed clinical trials comparing UKA vs. TKA for knee OA. The study reported that UKA had better functional outcomes and shorter surgical durations but also noted a higher risk of revision compared to TKA (51). Other systematic review compared UKA and TKA, focusing on operative time, blood loss, length of hospital stays, and postoperative outcomes. The study concluded that UKA was superior in early postoperative outcomes but had a higher long-term revision rate (52). The current study offers several advantages and new findings compared to these reviews: (1) unlike some previous reviews that focused on short-term outcomes, this study evaluates long-term outcomes over five years, providing more comprehensive insights into the durability and sustained benefits of UKA and TKA. (2) This study includes KSS, KSFS, ROM, and survival rates. This holistic approach offers a more complete assessment of the comparative effectiveness of UKA and TKA. (3) The study includes data from randomized controlled trials, cohort studies, and registries, enhancing the robustness and generalizability of the findings by capturing a broader spectrum of clinical practice and patient populations.

To reduce bias and address confounding factors such as population differences, surgical techniques, and post-operative care in studies comparing long-term outcomes of UKA and TKA, researchers can employ several methodological approaches. RCTs are ideal for evenly distributing confounders between groups, while multivariate regression analysis and propensity score matching can adjust for these factors statistically. Standardizing surgical techniques and postoperative care protocols, along with stratified analyses and sensitivity analyses, help ensure consistency and assess robustness. Comprehensive data collection, longitudinal follow-up, and meta-analyses of individual patient data (IPD) further enhance the accuracy and generalizability of the results, providing a clearer understanding of the true comparative effectiveness of UKA and TKA.

### 4.1 Potential clinical applications

The findings from this systematic review highlight the feasibility of implementing both UKA and TKA in clinical practice for managing knee osteoarthritis. The evidence indicating no definitive superiority of TKA over UKA in terms of pain relief and KSS over extended follow-up periods suggests that both surgical options are viable for long-term management. The trend favoring UKA in KSFS and ROM can guide clinicians in tailoring treatment plans more effectively. Patients who prioritize functional outcomes and have lifestyle or occupational demands requiring greater knee flexibility might benefit more from UKA. Conversely, TKA may be recommended for patients where longevity and durability of the implant are paramount, given the procedure's demonstrated long-term survival rates.

Possible side effects from UKA and TKA include infection, blood clots, implant loosening, and wear. UKA might also present risks such as bearing dislocation and progression of arthritis in other knee compartments, while TKA can involve more extensive bone removal, leading to longer recovery times and potential for greater post-operative pain. Management of these side effects includes meticulous surgical techniques to minimize infection risk, prophylactic anticoagulation to prevent blood clots, regular follow-ups to monitor implant stability, and physical therapy to enhance recovery and function. In cases of implant loosening or wear, revision surgery might be necessary. Patient education on signs of complications and adherence to post-operative care protocols are crucial in mitigating these risks.

To control the homogeneity of the subject population and achieve balance and equality in clinical studies, researchers should employ randomization to evenly distribute confounding variables across study groups. Stratified random sampling ensures that subgroups, such as age, gender, or severity of osteoarthritis, are proportionally represented. Matching subjects based on key characteristics before randomization can further enhance group comparability. Additionally, clearly defined inclusion and exclusion criteria help to create a uniform study population. Employing statistical techniques like propensity score matching during analysis can adjust for any remaining differences. Ensuring comprehensive data collection on all relevant variables and maintaining rigorous protocols for patient recruitment and follow-up are also critical in achieving balanced and equitable study groups.

### 4.2 Study limitations

Our study on the indications for UKA and TKA acknowledges the relevance of the scientific question, especially given the evolution in methodologies and patient outcomes over the past decade. The analyzed studies span from 10 to 20 years ago, highlighting a crucial limitation: the findings may not fully represent current medical practices and advancements in surgical techniques or patient management. Clinical guidelines have significantly shifted, emphasizing precise patient selection, surgical accuracy, and postoperative care, which heavily influence outcomes and effectiveness. Recent advancements, such as robotic-assisted surgeries, enhance the accuracy of implant placement in UKA, potentially leading to better outcomes and fewer revision surgeries compared to older methods. The trend towards personalized medicine, adapting procedures to individual patient anatomy and activity levels, challenges the broader applicability of older study conclusions. Methodological heterogeneity, publication bias, exclusion of large database studies, and language restrictions are notable limitations, introducing variability that affects comparability and generalizability. High heterogeneity in pain and ROM outcomes necessitates further investigation, considering patient demographics, surgical techniques, and postoperative care variations. The limited availability of long-term data poses challenges in understanding the sustained benefits and potential complications of UKA and TKA, underscoring the need for more comprehensive studies. These limitations necessitate cautious interpretation of results and emphasize the importance of including diverse data sources and minimizing methodological discrepancies in future research.

## 5 Conclusions

This systematic review highlights the nuanced outcomes of UKA and TKA for knee osteoarthritis over extended follow-up periods. While both procedures showed no clear superiority in terms of pain and KSS, UKA demonstrated better functional outcomes and ROM. At a five-year follow-up, TKA had an average survival rate of 97.5% compared to 90% for UKA, with TKA maintaining 89% survival at 15 years vs. 70% for UKA. These findings suggest TKA's superior long-term durability. Despite UKA's advantages in KSFS and ROM, TKA's higher survival rates indicate greater reliability and longevity, advocating for a personalized approach in surgical decision-making. Future research should address current limitations by incorporating balanced data sets and exploring technological advancements’ impact on patient outcomes.

## 6 Future research directions

Future research should focus on the long-term outcomes beyond five years to understand these surgical interventions’ enduring effects. Studies should evaluate long-term clinical and functional impacts, including survivorship, complication rates, and sustained quality of life improvements, such as pain scores, KSS, and ROM. Advanced methods like standardized outcome measures and stratified analyses are necessary due to the high heterogeneity in pain and ROM outcomes. Incorporating technological advancements such as robotic-assisted surgeries and personalized medicine can refine surgical techniques and improve patient-specific outcomes. These comprehensive, methodologically rigorous studies are essential for optimizing patient outcomes, guiding surgical practice, and informing patients about realistic expectations, ultimately leading to more personalized and effective treatment strategies for knee osteoarthritis. To address confounding variables like age and gender, multivariable regression models and propensity score matching will be used to ensure unbiased outcome comparisons between UKA and TKA patients.

## Data Availability

The original contributions presented in the study are included in the article/Supplementary Material, further inquiries can be directed to the corresponding author.

## Supplementary material

Supplementary Table S1 The baseline characters of included study.

Supplementary Table S2 Risk of bias table for random control trials using Cochrane collaboration's tool.

Supplementary Table S3 Risk of bias for cohort studies using Newcastle-Ottawa Scale.

Supplementary Table S4 Risk of bias for registry studies using Newcastle-Ottawa Scale.

Supplementary Table S5 The changing trend of KSFS and ROM between TKA and UKA groups.

Supplementary Table S6 PRISMA Checklist.
