# Supplementary material for: Comparative long-term outcomes of unicompartmental and total knee arthroplasty in knee osteoarthritis patients: a systematic review and meta-analysis
Source: Front Surg. 2024 Aug 21;11:1405025. doi: 10.3389/fsurg.2024.1405025 (PMC11371575; doi:10.3389/fsurg.2024.1405025)
Supplement: Supplementary Table S6 — PRISMA Checklist. [file Datasheet1.zip › Data Sheet 1_v1/SupMaterial/Table S2.DOCX]

Supplementary table S2. Risk of bias table for random control trials using Cochrane collaboration’s tool.

| Study Title | Sequence Generation | Allocation Concealment | Blinding | Incomplete Outcome Data | Selective Outcome Report | Free of Other Bias |
| --- | --- | --- | --- | --- | --- | --- |
| Costa CR, et al 2011 | Unclear (U) | ✅ | ✅ | ✅ | ❌ | ✅ |
| Newman J et al 1998 | ❌ | ✅ | ✅ | ❌ | ✅ | ✅ |
| Newman J et al 2009 | ❌ | ✅ | ✅ | ✅ | ❌ | ✅ |
| Weal et al 1999 | ❌ | ✅ | ✅ | ✅ | ✅ | ❌ |
| Beard et al 2019 | ✅ | ✅ | ✅ | ✅ | ✅ | ✅ |

✅ = Clear documentation that the study meets this requirement ❌ = No evidence that the study meets this requirement U = Unclear from publication. This table lists the findings related to the risk of bias in the studies mentioned as per the Cochrane collaboration’s tool.
