# Supplementary material for: Comparative long-term outcomes of unicompartmental and total knee arthroplasty in knee osteoarthritis patients: a systematic review and meta-analysis
Source: Front Surg. 2024 Aug 21;11:1405025. doi: 10.3389/fsurg.2024.1405025 (PMC11371575; doi:10.3389/fsurg.2024.1405025)
Supplement: Supplementary Table S6 — PRISMA Checklist. [file Datasheet1.zip › Data Sheet 1_v1/SupMaterial/Table S3.DOCX]

Supplementary table S3. Risk of bias for cohort studies using Newcastle-Ottawa Scale

| Study ID | Selection (1) | Selection (2) | Selection (3) | Selection (4) | Comparability (1) | Outcome (1) | Outcome (2) | Outcome (3) |
| --- | --- | --- | --- | --- | --- | --- | --- | --- |
| Bini et al 2017 | a | a | a | a | b | a | a | d |
| Gioe 2007 | a | a | a | a | b | a | b | b |
| Koskinen et al. 2008 | a | a | a | a | b | a | a | d |
| Liddle et al. 2014 | a | a | a | a | b | a | a | c |
| Lygre et al. 2010 | a | a | a | a | b | a | b | b |
| Niinimaki et al 2014 | a | a | a | a | b | a | a | c |
| Pearse AJ et al 2010 | a | a | a | a | b | a | b | c |
| Robertsson et al. 2009 | a | a | a | a | b | a | b | d |
| Martino et al 2020 | a | a | a | a | b | a | a | b |
| Hunt et al 2021 | a | a | a | a | b | a | b | c |
| Mohammad et al 2022 | a | a | a | a | b | a | b | c |
| Ackroyd et al. 2002 | a | a | a | a | b | c | a | b |
| Amin et al 2006 | a | c | a | a | b | c | a | d |
| Craik et al 2015 | a | c | a | a | b | c | a | d |
| Fabre Aubrespy 2016 | a | b | a | a | b | a | b | a |
| Horikawa et al. 2015 | a | b | a | a | b | c | a | a |
| Lum et al. 2016 | a | c | a | a | b | c | a | a |
| Lyons et al 2012 | a | b | a | a | b | c | a | d |
| Van der List et al. 2017 | a | b | a | a | b | c | a | b |
| Tan et al. 2021 | a | a | a | a | b | a | a | b |
| Brilliant et al. 2023 | a | a | a | a | b | a | b | b |
| Ma et al 2023 | a | a | a | a | b | a | b | c |
| Sessa and Celentano 2020 | a | a | a | a | b | a | a | a |

This table provides a detailed assessment of each study's risk of bias according to the categories defined by the Newcastle-Ottawa Scale for cohort studies. The 'a', 'b', 'c', and 'd' entries correspond to the specific ratings, across various domains including selection, comparability, and outcome.
